# Silicone oil complications in vitreoretinal surgery

**DOI:** 10.22336/rjo.2022.55

**Published:** 2022

**Authors:** Corina Cristina Coman (Cernat), Mihnea Munteanu, Stella Ioana Patoni (Popescu), Ovidiu Mușat

**Affiliations:** * Department of Ophthalmology, “Victor Babeș” University of Medicine and Pharmacy, Timișoara, Romania; ** Department of Ophthalmology, “Dr. Carol Davila” Central Military Emergency University Hospital, Bucharest, Romania

**Keywords:** silicone oil, posterior vitrectomy, complications

## Abstract

**Objective:** To illustrate the complications in subjects who experienced posterior vitrectomy and internal tamponade with silicone oil.

**Design:** Prospective, observational, longitudinal, descriptive, series of cases.

**Material and methods:** Patients who underwent posterior vitrectomy and internal tamponade with silicone oil of 1000 centistokes, from April to October 2021, were considered. All subjects included in the study had a complete ophthalmic examination pre and postoperatively on first day, first week and 1 to 6 months, and were assessed the best corrected visual acuity, ocular tension (raised > 21 mmHg, hypotony < 5 mmHg), emulsification, keratopathy, cataracts and posterior pole.

**Results:** 40 eyes of 40 patients were considered; twenty women and 20 men, with an average age of 63 years (range 46 to 77 years).

**Conclusions:** Complications are brief, treated medically or surgically, with better prognosis.

## Introduction

In the most recent years, the advance in vitreoretinal surgery has gone side by side with the advancement of replacers for vitreous, such as silicone oil, expansible gases with prolonged absorption and perfluorocarbon liquids [**1**].

In the beginning of the sixties, Dr. Paul Cibis initiated the implantation of silicone oil in the corpus vitreum of animals to supply permanent support to the retina, but anatomical and functional outcomes were not as anticipated [**2**]. In the 1970s, Scott propagated the use of silicone oil in numerous European centers, rethinking its use; nevertheless, the improvement of vitreoretinal procedure with silicone oil utilization was conducted until the works of Zivojnović [**3**,**4**]. Recently, silicone turned out to be an essential tool in managing very difficult situations of vitreoretinal surgery [**5**].

Various silicone oils are utilized in clinic and are composed of the similar molecule, polydimethylsiloxane [**6**]. Essentially, there are two ranks of viscosity: among 1,000 and 5,000 centistokes (cs), which are basically free from short chains [**7**]. In its perfect shade, it is chemically inactive, entirely penetrable to light and visual spectrum, can be decomposed, non-carcinogenic and simple to sterilize because it is resistant to heat [**7**]. 

Silicone oil is entirely transparent, has an index of refraction of 1.404 and is shade lighter than water; it is moisture permeable to oxygen and has an elevated surface tension, as compared with air and water [**8**]. Due to its refractive index, the optics of an eye that is full with silicone oil transforms: in the phakic eye, the concave anterior surface of the silicone oil in contact with the lens causes a hyperopic change of up to +5.00 diopters, while in the aphakic eye the convex surface of silicone oil causes a change of up to -5.00 diopters [**9**]. Because of its optical properties along with its moderate refractive changes, visual recovery is faster and laser can be utilized easier in retinal injuries or in diabetic retinopathy [**10**].

The action modes of silicone oil in the eye can be outlined as it follows:

• Intraocular tamponade: because of its high surface tension, silicone oil is capable to tamponade any retinal imperfection in all the parts of the retina regardless of size; unaccompanied vitreoretinal traction can keep it applied [**11**].

• Space filling: considering the stability of the silicone bubble and its property of not mixing with water, it limits free movement of proliferative cells and chemical mediators into the vitreous cavity and compartmentalizes the eye again in such manner that it can stop the progress of a new proliferative vitreoretinopathy [**12**]. Filling of space can prevent the factor from diffusing angio-proliferative in diabetic retinopathy [**12**].

• Mechanical repression of membrane shrinkage: the silicone oil bubbles inside the eye produce the traction forces to be oriented parallel to the retina, changing the traction vectors [**13**].

• Hemostasis: silicone oil limits the presence of blood and fibrin in the space between the retina and the bullae of the silicon, diminishing the proliferative effect; in this manner, it can play a role in limiting the development of rubeosis iridis in proliferative diabetic retinopathy [**13**].

• Prevention of phthisis: silicone slows down the process of phthisis and preserves the volume of the eye [**13**].

The objective of applying silicone oil during pars plana vitrectomy is to promote the release of retinal traction and retinal relocating on the retinal pigment epithelium [**14**]. As already described, present indications for silicone oil use are the following [**14**]:

• Restoration of retinal detachment due to giant tear [**14**].

• Cases of advanced proliferative vitreoretinopathy [**15**].

• Tractional retinal detachments in diabetic or mixed retinopathy, or cases of failed vitrectomy [**16**].

• Retinal detachments secondary to penetrating trauma with proliferative vitreoretinopathy [**17**].

Silicone oil is utilized in the end of the surgical intervention, when the breakdown of the membranes has already been done, closing all injuries and releasing traction forces [**18**]. It is applied after the fluid-gas exchange and between 3 and 4 cubic centimeters of oil are injected [**18**]. Silicon of 1000 or 5000 cs is brought to the level of the iris plane [**18**]. In the postoperatively period, the subject should be placed in the dorsal position to avoid the reach of the oil to the cornea inside [**18**]. Silicone use has been related with serious adverse reactions such as glaucoma, recurrent retinal detachment, keratopathy and hypotony; incidence of these complications differs considerably (8-40%) and modern studies record higher incidences than originally reported in Silicone oil studies [**19**-**21**].

Obviously, complications of the underlying disease can happen and can be determined by the technique used and altered intraocular physiology and, last but important, the properties and purity of the silicone oil applied can arise because of the existence of silicone oil in the preretinal space (cataract) or in the anterior segment (glaucoma, keratopathy), occurring more in both phakic and aphakic eyes [**22**].

Considering that inconstant percentages of presenting adverse reactions are reported due to the use of silicone oil in vitreoretinal surgery, it is significant to prospectively analyze their presentation to determine which factors can be modified for prevention [**22**]. 

## Objective

To detail the postoperative complications observed in patients undergoing pars plana vitrectomy combined with the silicone oil tamponade.

## Material and method

A prospective, longitudinal, observational, descriptive, case series study was undergone in the Ophthalmology Department of “Dr. Carol Davila” Central Military Emergency University Hospital, Bucharest, from April to October 2021.

All subjects who suffered from pars plana vitrectomy combined with silicone oil tamponade were included, regardless of age and gender.

Patients with corneal opacity who did not allow the adequate visualization of the posterior pole and patients who were not compliant with the tracing were excluded from the study.

Pre- and postoperative evaluation included of a complete ophthalmologic examination with visual acuity, applanation tonometry, slit-lamp biomicroscopy and indirect ophthalmoscopy.

Follow-up was performed on the first postoperative day, the first week, and from the first to the sixth month, because the complications from the use of silicone could start to be noticed as early as the first week postoperatively and, according to the specialized literature, the percentage increases every month [**22**]. Most reports consider the 6-month postoperative review to be the standard for determining ophthalmologic alterations, as retinal lesions are usually stable at 6 months [**22**].

According to the aim of the study, ocular hypertension was defined as an intraocular pressure > 21 mmHg or > 20 mmHg with antiglaucoma medication, measured at any time during the follow-up and hypotony as intraocular pressure < 5 mmHg [**23**].

Keratopathy was determined as the presence of bullae, band keratopathy, stromal or epithelial edema, or located opacities [**24**]. Emulsification was delimited as either silicone oil drop noted in the anterior chamber or by gonioscopy [**24**]. Cataract was defined as any opacity in the developed lens at any time postoperatively [**25**]. All subjects experienced a 3-port, 25-gauge pars plana vitrectomy with 1000 centistokes silicone oil tamponade.

Forty eyes of 40 subjects were analyzed, 20 of females (50%) and 20 of males (50%), with the mean age of 63 years (range 46 to 77 years), the right eye being affected in 21 patients (60%) and the left eye in 19 (40%). The diagnosis of 17 patients (42.5%) involved tractional or mixed retinal detachment secondary to advanced proliferative diabetic retinopathy. 17 patients (42.5%) had rhegmatogenous retinal detachment (complicated with proliferative vitreoretinopathy greater than C3 in 10 patients, redetachment in 7 patients). 2 patients had giant tear, 2 patients were registered with penetrating eye trauma (5%), 1 patient with macular hole (2.5%) and 1 with endophthalmitis (2.5%).

4 patients underwent further surgical procedures to vitrectomy combined with silicone oil: 2 phacofragmentation, 2 lensectomy and finally phacoemulsification with the placement of the intraocular lens. 

Regarding the 40 patients, 30 concluded the follow-up at 3 months, evaluating visual acuity in this group. Preoperatively, 10 patients were found with a visual acuity to count fingers, 9 to hand movement, 7 at 20/ 200-20/ 400, 3 to light perception and 1 at 20/ 80. Postoperatively, 11 patients were found at 20/ 200-20/ 400, 10 to counting fingers, 5 to hand movement, 2 to non-perception of light (one of them with preoperative diagnosis of endophthalmitis and the other with severe eye trauma), 1 to light perception and the 20/ 80 patient maintained the same visual ability.

In our study, thirty-six patients remained phakic, and by the sixth postoperative month, 15 developed a certain degree of lens opacity. Twenty-two patients developed ocular hypertension, this being observed from the first postoperative day. Two of our patients presented with hypotony (patients with visual acuity without light perception).

Four patients, 2 pseudophakic, 1 aphakic and 1 phakic, presented silicone in the anterior chamber. In nine patients (22.5%), retinal redetachment was observed, located in the lower sectors, and one patient (2.5%) developed subretinal fibrosis in the macular area.

## Discussion

Most series recorded retinal detachment complicated by proliferative vitreoretinopathy greater than C3 as the main indication for the use of silicone oil in vitreoretinal surgery. In the current study, the major indication was tractional detachment or mixed retinal detachment secondary to advanced proliferative diabetic retinopathy. This is because our population is abundant in diabetic patients who end up with advanced proliferative diabetic retinopathy [**26**].

The visual acuity of patients undergoing vitrectomy, combined with silicone oil tamponade, settles towards the third postoperative month [**27**]. The current study provided an apparent improvement; however, we must bear in mind that the final visual acuity depends on diverse factors such as preoperative conditions, number of surgical procedures performed, intraoperative findings, and postoperative complications [**27**]. In our study, no patient suffered silicone oil removal, so inconclusive visual acuity presented. Two of the patients ended up with no light perception, because one of them had endophthalmitis and the other severe eye trauma. The preoperative visual capacity of 20/ 80 of one of the patients preserved until the third postoperative month.

Early postoperative elevation of intraocular pressure can be observed in 7% to 48% of the eyes and is associated with inflammation, pupillary block, migration of silicone into the anterior chamber with consequent mechanical limitation of aqueous humor flow and preexisting glaucoma [**28**].

Infiltration of the trabecular meshwork by silicone droplets is believed to be the most important cause for the development of glaucoma [**28**]. When you get a drop in your eyes, the silicone is so large that it moves the iris, and it may as well close the angle and increase the pressure [**28**].

This rise is generally well suppressed with topical antiglaucoma drugs and is usually convertible after removal of the silicone oil [**28**].

In the first weeks, the causes for the rise in intraocular pressure during the time that the silicone is in the vitreous cavity are preexisting open-angle glaucoma and silicone oil emulsification [**28**]. 

After applying the silicone oil, the circumstances that can rise the intraocular pressure are the formation of drops in the anterior chamber or the locking of the lower iridectomy. At the end, the main causes are oil emulsification or pre-existing glaucoma [**28**].

Ocular hypertension is known in literature as the second complication from using silicone oil [**29**]. In our series, it represented the first complication (45.8% of patients). It was introduced from the first postoperative day, observing a decrease in intraocular pressure in the first 2 months and steadiness towards the third part of the postoperative period. To control this hypertension, 50% of these patients required a local hypotensive, 18.2% required 2 topical hypotensive drugs, and 31.8% required 3 topical hypotensive drugs. From this last group, 3 patients required acetazolamide support administered orally for 1 week. Treatment began with a beta-blocker and later a carbonic anhydrase inhibitor and finally a prostaglandin analogue was included.

Cataract is the most prevalent complication of the application of silicone oil in the human eye, showing mostly as posterior subcapsular opacity [**30**]. Long-term trials show a high prevalence of up to 90 to 100% of the patients, with peak presentation between 6 and 18 months after surgery [**31**]. Some authors underline that lens changes occur early. There are miscellaneous theories of development of cataract in these subjects [**31**]. Scott states that the metaplasia of the squamous cell epithelium of the lens appears as part of the proliferative response, the proof being the patients with complicated retinal detachments [**32**]. Other authors indicate that the combination of silicone oil with the posterior capsule of the lens causes toxicity and modifies its normal metabolism [**33**]. Histopathological studies prove that there is no silicone within the lens in very dense opacities [**33**]. In these patients, silicone removal is known to delay, but not prevent, the formation of cataracts, because 100% of the patients had cataracts during a 2-year follow-up period [**33**].

Cataract progress was also found in specialized literature [**33**]. It is a well-known fact that 100% of the patients subjected to vitrectomy combined with silicone oil evolved to cataract in spite of its removal [**33**].

Persistent hypotension appears in up to 24% of the patients’ eyes [**34**] and the most predisposed cases are those with preoperative proliferative vitreoretinopathy [**34**]. This is probably the result of the association of vitreoretinopathy with cyclitis, traction and detachment of the ciliary body after decreased aqueous humor production, the result being the occurrence of hypotonia [**34**]. In our series, this adverse reaction was reported in 4% of the patients. 

Retinal reattachment is established inside the percentages stated in literature [**35**]. Considering that the positioning of the redetachment is in the lower sectors, it is desirable to keep these patients under close observation because it generally does not affect the macular area, which it is protected by silicone oil [**35**].

The silicone in the anterior chamber was presented as a result of deficiency of patient positioning. Two of them required surgical removal of silicone, without additional complications.

## Conclusions

It is widely known that silicone oil is a useful tamponade method, which permits early visual rehabilitation of the patient, along with additional procedures (for example: laser application).

Complications that come up from the use of silicone oil in vitreoretinal surgery are generally temporary and benefit from an adequate management, whether medical or surgical, that allows the patient to have a good long-term prediction.

The complications in our case series did not vary from the ones recorded in literature; nevertheless, longer follow-up is essential to estimate long-term complications.

In addition, regardless of the complications due to the use of silicone oil, the prevalence of which is comparatively low, the advantage to the patient is higher.

**Conflict of Interest statement**

The authors state no conflict of interest.

**Informed Consent and Human and Animal Rights statement**

Informed consent has been obtained from all individuals included in this study.

**Authorization for the use of human subjects**

Ethical approval: The research related to human use complies with all the relevant national regulations, institutional policies, is in accordance with the tenets of the Helsinki Declaration, and has been approved by the review board of “Dr. Carol Davila” Central Military Emergency University Hospital, Bucharest, Romania.

**Acknowledgements**

None.

**Sources of Funding**

None.

**Disclosures**

None.
